# Health Statuses of People in Poverty Receiving Public Assistance in Japan: A Scoping Review

**DOI:** 10.31662/jmaj.2024-0062

**Published:** 2024-06-28

**Authors:** Haruna Kawachi, Daisuke Nishioka

**Affiliations:** 1 Department of Medical Statistics, Research & Development Center, Osaka Medical and Pharmaceutical University, Takatsuki, Japan

**Keywords:** Poverty, Public assistance, Scoping review, Japan

## Abstract

**Background:**

Promoting health and well-being is essential to ensure dignified lives of the entire population, including those living in poverty. Guaranteeing the human right to health is a critical responsibility of social security policies. To address emerging issues associated with poverty, the Japanese government has implemented a welfare program known as public assistance―*seikatsu-hogo*. However, financial welfare programs may not fully mitigate health risks due to the complex impact of poverty on health. Although a global systematic review of the health status of public and social assistance recipients has been conducted, it did not include any studies from Japan. Furthermore, evidence for the development of health support strategies for Japanese recipients remains scarce. This scoping review aims to identify the current situation and potential issues concerning the health of recipients.

**Methods:**

PubMed was searched for articles published before November 2023. Of the 357 articles identified, 56 were included. Among those included, 35 used the individual status of receiving public assistance as an exposure variable, 13 considered public assistance recipients as the study population, and 8 used the prefectural proportion of the population receiving public assistance as an environmental predictor.

**Results:**

We found that public assistance recipients tend to have more disadvantageous health and well-being statuses than the general population, as reported in the global systematic review. Health inequalities were also observed among recipients based on their sociodemographic characteristics. In Japan, public assistance recipients face several health risks and are at a disadvantage compared with the general population.

**Conclusions:**

The distribution of risks is heterogeneous among recipients, despite the minimum income protection and financial benefits in health and long-term care use. Further studies to identify the effects of public assistance policy on the health of the impoverished population, evidence-based discussions, and reform of social security policies are warranted.

## Introduction

Promoting health and well-being are prerequisites for the dignified lives of the entire population, and guaranteeing the human right to health for the people is one of the important roles and responsibilities of society and social security policies ^(1), (2), (3)^. However, global evidence has recently indicated that people’s social backgrounds and contexts certainly inhibit their health-promoting activities and healthy lifestyles, resulting in health inequalities across their socioeconomic statuses ^(4), (5)^. Poverty is known to be a major, but unmet, social determinant of health. People living in poverty have insufficient resources to meet their health needs, which can impede cognitive function, resulting in difficulty making reasonable health investments and leading to adverse health outcomes ^(6), (7)^. Thus, it is imperative to reform the social security system to address the negative health effects of poverty.

In recent decades, Japan has been facing emerging issues associated with poverty. For example, Japan’s poverty rate was reported to be 15.4% in 2021, which is higher than the average among member nations of the Organization for Economic Cooperation and Development ^(8)^. In particular, the poverty rate among children living with single parents has been raised as a serious problem. Approximately half of children living with single parents live below the poverty line. The Constitution of Japan Article 25 stipulates that “All people shall have the right to maintain the minimum standards of wholesome and cultured living” and that “In all spheres of life, the state shall use its endeavors for the promotion and extension of social welfare, security, and public health ^(9)^.” As a measure to embody this clause, a governmental welfare program called public assistance―*seikatsu-hogo*―exists, which can benefit households living below the poverty line. The Municipal Welfare Office conducts rigorous means tests and determines whether people will receive benefits ^(10)^. Approximately 1.6% of the entire population in Japan was receiving public assistance in 2021. Households receiving public assistance can benefit from monthly minimum income benefits and are fully exempt from payments for healthcare and long-term care utilization ^(10)^.

Considering the potential influence of multidimensional poverty ^(11)^, financial welfare programs may not fully compensate for socioeconomic health risks. A previous review by Shahidi et al. reported that the health status of public and social assistance recipients in high-income countries is not better than that of nonrecipients ^(12)^. From the review, public or social assistance recipients have poorer subjective health; a higher prevalence of mental illness, including anxiety and depression, diabetes, and obesity; and a higher mortality rate than the general population not receiving public or social assistance. Furthermore, public and social assistance recipients have been reported to be more likely than nonrecipients to engage in high-risk health behaviors, such as smoking, problematic drinking, and frequent visits to healthcare institutions. Shahidi et al. summarized these findings and suggested that public and social assistance programs may not sufficiently protect the health of recipients.

Since the fiscal year 2021, from the perspectives of promoting health and optimizing medical assistance costs for public assistance recipients, a health support program for public assistance recipients―*kenko-kanri-shien-jigyo―*has been mandated in municipal offices. This program emphasized the importance of a data-driven approach. However, because public and social assistance systems vary across countries and the review by Shahidi et al. did not include any Japanese evidence ^(12)^, the generalizability of the evidence was unclear. In addition, quantitative data on health support strategies for recipients of public assistance in Japan remain sparse. Summarizing the existing evidence to develop evidence-based health support policies for recipients is urgently needed.

In this study, we aimed to conduct a literature review of existing peer-reviewed research that has dealt with the health and health behaviors of public assistance recipients in Japan. Considering the findings of this review, we further aimed to organize the evidence and summarize emerging issues that can contribute to the discussion of effective interventions to support health among recipients.

## Materials and Methods

### Study design

A scoping review.

### Eligibility criteria

We used the population, concept, and context framework to define the inclusion criteria. All published studies using public assistance recipients in Japan, both as an exposure variable and study population, were eligible. We reviewed the existing literature describing the health and well-being of public assistance recipients as an outcome measure. We restricted our search to English-language and peer-reviewed publications. We excluded gray literature, working papers, and peer-reviewed commentaries lacking direct empirical tests.

### Search strategy

We conducted a search on PubMed using the term ((“public assistance” [MESH] or “public assistance”) AND (Japan)) on November 7, 2023. We supplemented our electronic search by searching the reference lists of all the included literature and related review articles.

### Study selection

The studies were independently selected by two researchers (HK and DN). The two researchers compared their lists, and any differences in opinions were resolved through discussion.

### Data extraction and analytic strategy

Data were extracted by the authors using standard data extraction forms, including the PMID, title, author names, and publication year of the articles. Whether public assistance was used as an exposure variable or as a study population was coded. If a study utilized public assistance as an exposure variable, we extracted the study design, setting, reference group information, study participants, sample size, study outcomes, and study findings. If the study utilized public assistance recipients as the entire study population, the study design, setting, sample size, and findings were extracted.

## Results

**Figure 1.**
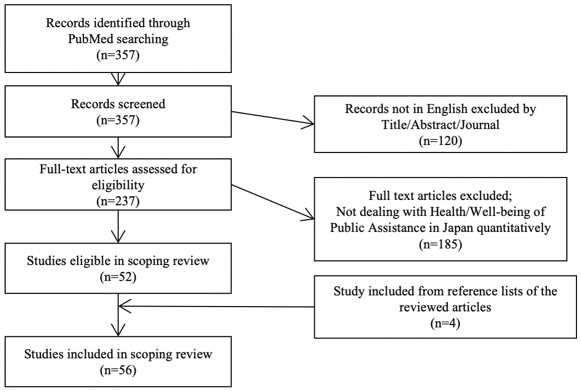
Flow diagram of the scoping review.

A total of 357 articles were identified in the search, of which 52 met the eligibility criteria of this review ^(13), (14), (15), (16), (17), (18), (19), (20), (21), (22), (23), (24), (25), (26), (27), (28), (29), (30), (31), (32), (33), (34), (35), (36), (37), (38), (40), (41), (42), (43), (44), (45), (46), (47), (48), (49), (50), (51), (52), (53), (54), (55), (56), (57), (58), (59), (60), (61), (62), (63), (64)^. From the review, four eligible studies were additionally included ^(65), (66), (67), (68)^. Finally, 56 articles were included. The selection process of the study is illustrated in Figure 1. There have been no randomized controlled trials on this study design.

### Studies using public assistance as an exposure variable

A total of 35 studies utilized public assistance status as the exposure variable ^(13), (14), (15), (16), (17), (18), (19), (20), (21), (22), (23), (24), (25), (26), (27), (28), (29), (30), (31), (32), (33), (34), (35), (36), (37), (38), (39), (40), (41), (42), (43), (44), (65), (66), (67)^. Among them, seven used public data obtained from national or local governments ^(17), (21), (22), (23), (27), (32), (38)^. One study used registry data obtained from medical institutes ^(35)^. Majority of the studies used data collected from medical records and/or administered surveys through questionnaires or interviews with healthcare providers at medical institutes. A higher mortality ^(67)^, prevalence of infections ^(24)^, chronic diseases (diabetes complications ^(19), (36)^ and heart diseases ^(30), (31), (35)^), and adverse health outcomes of surgeries ^(15)^ were observed. Unpreferable health behaviors were also prevalent among public assistance recipients such as smoking ^(31)^, frequent medical visits ^(25), (26), (38), (65)^, polypharmacy ^(40), (41)^, less vaccine uptake ^(42)^, and refraining from necessary healthcare-seeking behaviors ^(16), (27), (44)^. However, several studies have also demonstrated an association or no association between receiving public assistance and favorable health outcomes/behaviors ^(14), (17), (18), (28), (32), (34)^. In a multicenter prospective cohort study, Kaneko et al. investigated that the receipt of public assistance was inversely associated with all-cause mortality among patients who had started receiving regular physician-led health care at home or at nursing homes ^(28)^. Furthermore, among patients who were diagnosed with methamphetamine use disorder, those who received public assistance were more likely to remain in treatment at 3 months than those who did not (Table 1) ^(14)^.

**Table 1.** Descriptive Information of the Studies Using Public Assistance as an Exposure Variable.

Authors	Year	Study design	Setting/participants	Data	Reference group	N (public assistance (PA)/non-PA)	Outcome	Findings
Noda T, et al. ^(13)^	2001	Prospective cohort study	Patients with alcoholism attending to an abstinence self-help group	Medical records and a follow-up interview with each patient or their family	Nonrecipients	306 (83/214), 9 had no information on receiving PA	Stable abstinence from alcohol and mortality	Non-PA recipients have more preferable odds of stable abstinence from alcohol (OR: 7.2, 95% CI: 1.8-28.4) and lower mortality (HR: 0.5, 95% CI: 0.4-0.8) than PA recipients
Kobayashi O, et al. ^(14)^	2008	Case-control study	Single center/patients diagnosed with methamphetamine use disorder (aged 19-60 years)	Medical records	Nonrecipients	101 (26/75)	3-month treatment retention	The treatment retention group had a significantly larger proportion of patients receiving PA (38% vs. 17%). Those who received PA were twice as likely to remain in treatment as those without, although the *p*-value was statistically borderline (adjusted OR: 2.38, 95% CI: 0.92-6.16, *p* = 0.074)
Wada T, et al. ^(15)^	2009	Retrospective cohort study	Patients with lateral epicondylitis treated with arthroscopic surgery (aged 42-71 years)	Medical records and a self-administered questionnaire survey	Nonrecipients	18 (4/14)	Disability of the Arm, Shoulder, and Hand score	PA was found to be a potential predictor of a poorer outcome after arthroscopic surgery for lateral epicondylitis (median score: 36.5 vs. 2.2, *p* = 0.03)
Kamimura A, et al. ^(16)^	2013	Retrospective cohort study	Data were collected through semistructured, in-person interviews with women who had experienced any type of intimate partner violence (IPV) in their lives (aged 24-80 years)	Interview survey	Nonrecipients	3,403 person-years of 101 respondents, including 154 person-years of the recipients	Intimate partner violence-specific healthcare-seeking behavior	Receiving PA was associated with IPV-specific healthcare-seeking behavior (coef.: 1.08, SE: 0.48)
Uchimura K, et al. ^(17)^	2015	Retrospective cohort study	Newly registered tuberculosis (TB) patients aged 15-59 years in Japan	Nationally representative registry data	Other insurance	9,097 (711/uninsured: 321, insured: 8,065)	Tuberculosis death	The association between PA and death from tuberculosis disappeared in the multivariate model. However, the association remained among the uninsured (aHR [95% CI]: 1.08 [0.78-1.51] and 1.48 [1.02-2.15], for PA and uninsured, respectively). PA, particularly if limited to casual workers and unemployed persons, may contribute to the improvement of survival rates (from the Kaplan-Meier curve results)
Takenaka Y, et al. ^(18)^	2016	Retrospective cohort study	Single center/newly diagnosed and histologically confirmed patients with head and neck squamous cell carcinoma (HNSCC), median age 76 years (range 28-92)	Medical records	Other insurance	407 (72/335)	Mortality	The clinical stage distribution was not significantly different between the PA groups and other insurances (OR: 1.36, 95% CI: 0.78-2.39, for stages III and IV). The 5-year overall survival, cumulative incidence of HNSCC death, and cumulative incidence of other death rates were 63.3% and 59.1%, 27.0% and 31.8%, and 10.3% and 9.7% for the PA and other insurance groups, respectively. The adjusted subdistribution HR for the association between PA and HNSCC death was 0.73 (95% CI: 0.44-1.21)
Funakoshi M, et al. ^(19)^	2017	Cross-sectional study	Multicenter/young adult patients (aged 20-40 years) with type 2 diabetes (T2DM)	Medical records and a self-administered questionnaire survey	Other insurance	672 (64/608)	Diabetic complication	PA recipients have a higher odds ratio of having diabetes complications after adjusting for general risk factors (an OR of 2.19 (1.20-3.95) for retinopathy and an OR of 2.60 (1.16-5.50) for nephropathy
Kawahara YY, et al. ^(20)^	2017	Retrospective cohort study	Single center/patients admitted to the emergency room and then had an episode of self-harm	Medical records	Nonrecipients	405 (54/351)	Repetition of short-term self-harm (<1 month and <6 months)	Living on PA was associated with repetition of self-harm within 6 months (multivariable HR: 3.126 [1.699-5.754]).
Nakanishi M, et al. ^(21)^	2017	Panel data	National Patient Survey	Nationally representative survey data	Nonrecipients	13,014 (1,112/11,902)	Deliberate self-harm	The proportion of patients receiving PA was higher in those who had engaged in deliberate self-harm (8.5%) than in the general population
Yuda M. ^(22)^	2018	Cross-sectional study	- The Fact-Finding Survey on Medical Assistance (Iryo-Fujo Jittai Chosa). - The Survey of Medical Care Activities in Public Health Insurance (Syakai Iryo Shinryo-Koui Betsu Chosa)	Nationally representative sets of individual-level claims data	Other insurance	299,520 (18,693/280,827)	Inpatient care elasticity	The medical expenditure is significantly higher for medical assistance patients than for other medical health insurance patients, with an arc elasticity of approximately 0.20
Izumi K, et al. ^(23)^	2019	Panel data	One municipality/patients with culture-positive TB notified with genotype data of *Mycobacterium tuberculosis*	Registry data	Nonrecipients	1,025 (304/720), 1 had no information on receiving PA	Clustered cases	Receiving PA (adjusted OR: 1.81, 95% CI: 1.15-2.84) at the time of tuberculosis diagnosis was associated with genotype clustering
Cho T, et al. ^(24)^	2020	Retrospective cohort study	Single center/women who underwent *Chlamydia trachomatis* testing during a singleton pregnancy and delivered after the 22nd week of gestation (control group: randomly selected individuals from among patients with negative results)	Medical records	Nonrecipients	2,233 (74/2,159)	Preterm birth	The Chlamydia-positive group had a higher rate of PA coverage than the control group (12.4% vs. 2.1%)
Ikeda K, et al. ^(65)^	2020	Case-control study	Single center/patients who visited the Emergency Department (ED), aged ≥20 years	Medical records	Other insurance	340 (27/313)	Frequent ED use	The receipt of PA significantly increased the OR for frequent visits even after adjusting for sex, age, and potential confounding factors (OR: 3.89, 95％ CI: 1.62-9.35)
Kaneko M, et al. ^(25)^	2020	Cross-sectional study	Multicenter/patients who presented to the EDs	Medical records	Nonrecipients	20,388 (110/20,257), 21 had no information on receiving PA	Frequent ED use	Receiving PA (adjusted OR: 7.19, 95% CI: 2.87-18.07) had an association with frequent ED visits
Osawa I, et al. ^(26)^	2020	Cross-sectional study	Single center/ED patients (age ≥ 18 years)	Medical records	Nonrecipients	556 (37/519)	Hospitalization rates	Frequent ED users tended to receive PA more than nonfrequent ED users (8.9% vs. 4.7%). The risk for hospitalization was not associated with frequent ED use (adjusted OR: 1.21, 95% CI, 0.74-1.96)
Yoshikawa R, et al. ^(27)^	2020	Panel data	Japanese tuberculosis surveillance data	Registry data	Other insurance	88,351 (7,148/81,203)	Patient delay (the time from the onset of symptoms to the initial doctor visit): short, moderate, and long delay	Receiving PA was specifically a risk factor for moderate and long delays (adjusted OR [95% CI]: 1.19 [1.09-1.31] and 1.36 [1.16-1.60] for moderate and long delays, respectively)
Kaneko M, et al. ^(28)^	2021	Prospective cohort study	Multicenter/patients who had started receiving regular physician-led health care at home or at nursing homes, aged ≥65 years	Survey data, administered by physicians	Nonrecipients	825 (115/710)	All-cause mortality	A multivariate Cox proportional-hazards model showed that nonreceipt of PA was associated with mortality, indicating that patients who received PA were less likely to die, adjusted HR: 0.61 (0.41-0.90) with multiple imputation (complete case analysis, n = 663: 0.65 [0.42-1.01])
Koyama Y, et al. ^(29)^	2021	Cross-sectional study	One municipality/a survey was administered to caregivers of all 6-7-year-old children attending public elementary schools	Social survey data (self-questionnaire)	Low-income (LI) households without PA	6,920 (PA(+)/LI(+): 191, PA(−)/LI(+): 452, PA(−)/LI(+): 6,277)	Child mental health assessed using the Strength and Difficulties Questionnaire (SDQ) and child resilience assessed using the Children’s Resilient Coping Scale (CRCS)	The SDQ and CRCS scores were not significantly different between recipients or nonrecipients of PA. However, the mental health outcomes were worse in PA recipients across all scores, particularly emotional symptoms and prosocial behavior. Children in households receiving PA had a higher risk of school refusal than those in households not receiving PA; children living in PA household were four times more likely to refuse to go to school than children living in LI households without PA (OR: 4.00, 95% CI: 0.85-18.84, *p* = 0.080)
Watanabe S, et al. ^(30)^	2021	Retrospective cohort study	Single center/patients with ST-segment elevation myocardial infarction	Medical records	Nonrecipients	525 (67/458)	Clinical features	The prevalence of smoking was higher in the PA group than in the non-PA group (91.0 vs. 81.1%, *p* = 0.02). The high-density lipoprotein cholesterol level in the PA group was lower than that in the non-PA group (43.2 ± 9.9 mg/dL vs. 47.1± 12.8 mg/dL, *p* = 0.005). Ventricular arrhythmia on admission was significantly more frequent in the PA group than in the non-PA group (11.9 vs. 4.8%, *p* = 0.02). The left ventricular ejection fraction in the PA group was lower than that in the non-PA group (in the acute phase 46.6％ ± 10.7 vs. 53.3± 8.6, *p* = 0.001; in the chronic phase 48.7% ± 10.1 vs. 55.3 ± 9.4, *p* = 0.008)
Fujito H, et al. ^(31)^	2022	Retrospective cohort study	Single center/patients who were admitted for acute heart failure (HF) and were discharged or transferred to another hospital, followed up for 1 year	Medical records	Other insurance	771 (87/684)	Cardiac events (death from cardiovascular disease (CVD) or readmission) after discharge	The PA group was significantly younger and had a higher incidence of diabetes, smoking, and ischemic and hypertensive heart disease as well as lower estimated glomerular filtration rate than the non-PA group (all *P* < 0.05). Patients with acute HF covered by PA received the same quality of medical care, including invasive therapy, as those not covered by PA After adjusting for covariates, PA was independently associated with 1-year cardiac event rate (HR: 2.15, 95% CI: 1.42-3.26)
Kaneko M, et al. ^(32)^	2022	Cross-sectional study	One municipality/older adults aged 75 years or older who visited medical facilities at least four times a year	Claims data	Nonrecipients	413,600 (14,243/399,357)	Care fragmentation (Fragmentation of Care Index (FCI))	Multivariable analysis revealed that patients receiving PA had a lower FCI than those not receiving PA, with a coefficient of 0.137 (This can be explained by the fact that people receiving PA need to report to the local government the name of the medical institution where they would like to visit.)
Kino S, et al. ^(33)^	2022	Cross-sectional study	39 municipalities/older community-dwelling recipients	Social survey data (self-questionnaire)	Nonrecipients	93,280 (1,093/92,187)	Depressive symptoms (Geriatrics Depression Scale GDS)	The older PA recipients have a higher prevalence of depressive symptoms (PR 1.57 [95% CI: 1.47-1.67]) than nonrecipients
Kino S, et al. ^(66)^	2022	Cross-sectional study	39 municipalities/older community-dwelling recipients	Social survey data (self-questionnaire)	Nonrecipients	16,135 (202/15,933)	Suicidal ideation and attempts	PA recipients had a higher prevalence of lifetime suicidal ideation (PR: 1.47, 95% CI: 1.02-2.13) and a higher prevalence of attempted suicide (PR: 1.91, 95% CI: 1.20-3.04) than nonrecipients
Nakayama T, et al. ^(34)^	2022	Case-control study	Single center/patients who initiated maintenance dialysis (median age (IQR) 70 (59-79) years)	Medical records	Nonrecipients	355 (13/342)	Peritoneal dialysis (PD) selection	Multivariate analysis revealed that PA (OR: 0.70; 95% CI: 0.08-6.09, *p* = 0.74) was not significantly associated with PD selection
Nishimoto Y, et al. ^(35)^	2022	Retrospective cohort study	Multicenter/patient with acute HF (AHF) and patients who were hospitalized because of AHF for the first time	Registry data	Nonrecipients	3,728 (218/3,510)	Cumulative 1-year incidences of all causes of death, HF, and hospitalizations after discharge	The adjusted risk for HF hospitalization beyond 180 days was significant in those with PA (HR: 1.56, 95% CI: 1.07-2.29, *p* = 0.02)
Sengoku T, et al. ^(36)^	2022	Cross-sectional study	People residing in Japan during the years 2015-2017	National Database (NDB) and Medical Assistance Claims data	Nonrecipients	The total numbers of recipients in Japan during 2015, 2016, and 2017 were 2,161,442, 2,148,282, and 2,130,482, respectively	Type 2 diabetes prevalence	The mean crude prevalence and age-standardized prevalence of diabetes (inpatients and outpatients) among 47 prefectures were 7.8% in recipients and 3.9% in public health insurance enrollees. In the city-level analysis, the odds ratio for the prevalence of T2D by region ranged from 0.31 to 1.51
Wakata S, et al. ^(37)^	2022	Repeated cross-sectional study	Patients in a single center clinic	Clinic medical records	Nonrecipients	374 (68/306)	The Health-Related Quality of Life by SF-12 score, with three components: physical health component summary (PCS), mental health component summary (MCS), and role-social component summary (RCS)	The PA recipients had lower PCS and RCS scores than those not receiving any welfare benefit (PCS Beta: −8.24, 95% CI: −10.43 to −6.05; RCS Beta: −7.87, 95% CI: 11.88 to −3.85). A decline in the MCS score was observed more in PA recipients than in those not receiving any welfare benefits during the COVID-19 pandemic (Beta: −4.27, 95% CI: −6.67 to −1.82)
Yuda M. ^(38)^	2022	Cross-sectional study	- The Fact-finding Survey on Medical Assistance for PA patients - the Survey of Medical Care Activities in Public Health Insurance for the universal public health insurance patients	Nationally representative sets of individual-level claims data	Other insurance	1,698,857 (261,546/1,437,311)	Outpatient care utilization	PA assignment increases monthly healthcare expenditure by approximately 20% and the monthly number of doctor visits by approximately 25%. When imposing a copayment on PA beneficiaries, monthly healthcare expenditure significantly decreases by approximately 25.0% and the number of visits by approximately 30%. The estimated price elasticity based on these results is extremely small, approximately −0.02, indicating that the level of copayment rate has a negligible effect on the intensive margin of outpatient healthcare utilization
Lu Y, et al. ^(39)^	2023	Prospective cohort study	19 municipalities/people aged ≥65 years who were not certified as requiring long-term care	Social survey data (self-questionnaire)	Nonrecipients	73,262 (Not available)	Functional disability over 5 years	In the ridge regression model, the characteristic of households receiving PA was an important predictor of functional disability
Miyake H, et al. ^(40)^	2023	Cross-sectional study	Single center/patients with rheumatoid arthritis	Survey data, administered by healthcare providers to the patients or their representatives	Nonrecipients	991 (17/974)	Polypharmacy and excessive polypharmacy	Excessive polypharmacy, defined as regularly taking 10 or more orally administered medications, was associated with the presence of PA (OR: 3.80, 95% CI: 1.23-11.72).
Miyake H, et al. ^(41)^	2023	Cross-sectional study	Single center/patients with systemic lupus erythematosus (SLE)	Survey data, administered by healthcare providers to the patients or their representatives	Nonrecipients	261 (8/253)	Polypharmacy and excessive polypharmacy	Excessive polypharmacy was associated with the presence of PA (multivariable OR: 18.9, 95% CI: 3.30-102.65)
Miyake H, et al. ^(42)^	2023	Cross-sectional study	Single center/patients with rheumatoid arthritis	Survey data, administered by healthcare providers to the patients or their representatives	Nonrecipients	991 (17/974)	Vaccination	Influenza vaccine: PA was significantly associated with nonvaccination
Nakamura Y, et al. ^(43)^	2023	Case-control study	Single center/those who visited the Department of Psychiatry and Neurology	Medical records	Never received PA	536 (87/449)	Medical visit behavior as a proxy for medication adherence	History of PA receipt was associated with higher nonattendance (OR: 2.04, 95% CI: 1.22-3.43, *p* = 0.007)
Kino S, et al. ^(44)^	2024	Cross-sectional study	60 municipalities/older community-dwelling recipients	Social survey data (self-questionnaire)	Nonrecipients	16,366 (229/16,137)	Dental visits	In the fully adjusted model, PA recipients were 24% less likely to have dental visits for any reason (prevalence ratio [95% CI], 0.76 [0.64-0.90]), 23% less likely for treatment (0.77 [0.65-0.92]), and 21% less likely for prevention [0.79 (0.65-0.95])
Kushibuchi M, et al. ^(67)^	2024	Retrospective cohort study	Single center/patients diagnosed with alcoholic liver cirrhosis	Medical records	Nonrecipients	244 (62/182)	All-cause mortality	The overall mortality rates were 48.4% and 31.9% for PA and non-PA recipients, respectively (*p* = 0.002). In the Cox regression model adjusted for age, ALBI score, HCV infection, and presence or absence of a designated key family contact, the hazard ratio for PA status was 1.75 (95% CI: 1.03-2.98, *p* = 0.039)

### Studies using public assistance as an entire study population

A total of 13 studies used public assistance recipients as their study population ^(45), (46), (47), (48), (49), (50), (51), (52), (53), (54), (55), (56), (68)^. Among them, one study used data from the Japanese national database, which identified regional differences in the prevalence of admission length in psychiatric hospital ^(45)^ by public assistance recipients and its predictors. Furthermore, eight studies used data from public assistance databases of the welfare offices of local governments ^(46), (48), (49), (50), (51), (53), (55), (56)^. Among the public assistance recipients, several characteristics were associated with undesirable health statuses and behaviors. For example, those living alone or unemployed were more likely to have diabetes and frequently attend medical consultations ^(46), (49), (51), (53)^. Four studies used data obtained from a self-questionnaire social survey ^(47), (52), (54), (68)^. The health behavior scale of older recipients living alone, who have the most disadvantageous health risks among recipients, was developed ^(47)^ and examined for validity ^(54)^. This scale is reportedly useful in predicting the health behaviors of older recipients living alone, such as health checkup ^(47), (54)^. High activity of daily living and social capital were found to be associated with the initiation and termination of public assistance (Table 2) ^(52), (68)^.

**Table 2.** Descriptive Information on the Studies Using Public Assistance as an Entire Study Population.

Authors	Year	Design	Settings/participants	Data	N	Outcome	Findings
Okumura Y, et al. ^(45)^	2019	Cross-sectional study	All PA recipients hospitalized in psychiatric hospitals	National database	46,559	Age-sex-standardized claim ratio of the psychiatric admission	There is a geographical (prefectural) variation in the number and total medical cost of psychiatric admissions among recipients. There is a positive correlation between recipients’ psychiatric admission, the number of prefectural psychiatric beds per 100,000 population, and the prefectural proportion of the population receiving PA
Nishioka D, et al. ^(46)^	2020	Retrospective cohort study	Adult recipients in two municipalities in Japan (>=20 years old)	Municipal database	6,016	Frequent outpatient attendance	Recipients living alone had an incidence of 1.58 (95% CI: 1.05-2.39) compared with those not living alone. Recipients visiting private institutions had an incidence of 1.74 (95% CI: 1.20-2.52) compared with those visiting medical corporations
Isozaki A, et al. ^(47)^	2021	Cross-sectional study	Randomly sampled older recipients in Japan	Social survey data (self-questionnaire)	1,280	Health checkup behavior	The health behavior scale was developed, including two constructs (self-perception of personal power and practical skills for daily health), which correlates with recipients’ health checkup behavior
Nishioka D, et al.^(48)^	2021	Retrospective cohort study	Children in the household receiving PA in two municipalities in Japan (<=15 years old)	Municipal database	573	Children’s acute and chronic diseases	Among PA recipients, living in single parenthood is associated with a higher prevalence of the following: Asthma (IR: 1.62, 95% CI: 1.16-2.26) Allergic rhinitis (IR: 1.41, 95% CI: 1.07-1.86) Dermatitis and eczema (IR: 1.81, 95% CI: 1.21-2.7) Dental diseases (IR: 1.79, 95% CI: 1.33-2.42) An insignificant association was observed between single parenthood and children’s acute health conditions
Nishioka D, et al. ^(49)^	2021	Retrospective cohort study	Adult recipients in two municipalities in Japan (>=20 years old)	Municipal database	2,698 younger adults (20-64 years old) and 3,019 older adults (>65 years old)	1-year cumulative incidence of diabetes diagnosis	Among younger (20-64 years old) men, the incidence of diabetes diagnosis was higher among those who were: Unemployed (IR: 1.28, 95% CI: 0.85-1.91) Living alone (IR: 1.48, 95% CI: 0.96-2.29). However, no significant association was observed among young women and older recipients
Nishioka D, et al. ^(50)^	2021	Retrospective cohort study	Households receiving PA to rear children in five municipalities	Municipal database	4,893 households	Households’ healthcare costs	Government savings through income reduction were counterbalanced by increased medical expenditure among child-rearing individuals in poverty (i.e., a 50 USD reduction in cash benefits may lead to a 248.6 USD increase in healthcare costs per household [95% CI: 25.4-471.7])
Nishioka D, et al. ^(51)^	2021	Retrospective cohort study	Adult recipients in two municipalities in Japan (>=20 years old)	Municipal database	4,497	Dental care access	Recipients who were younger (IR: 0.87 [by 10 years old], 95% CI: 0.84-0.91), women (IR: 1.22, 95% CI: 1.08-1.38), immigrants (IR: 1.53, 95% CI: 1.16-2.01), and with mental disabilities (IR: 1.30, 95% CI: 1.08-1.56) may have greater accessibility to dental care. Living alone and employment are potential predictors of dental care access.
Kino S, et al. ^(68)^	2022	Retrospective cohort study	Older community-dwelling recipients	Social survey data (self-questionnaire)	347	Starting or leaving PA program	People with higher perceived mutual community help were 1.21 times (95% CI: 1.02-1.46) more likely to commence PA 3 years later than those who did not. PA recipients who felt community attachment to their resident community were 1.16 times more likely to give up PA 3 years later than those who did not (95% CI: 1.06-1.28). Similarly, those who had social roles were 1.15 times more likely to give up PA 3 years later than those who had not (95% CI: 1.01-1.30).
Kino S, et al. ^(52)^	2022	Retrospective cohort study	Older community-dwelling recipients	Social survey data (self-questionnaire)	335	Changes in social relationships	Recipients who stopped receiving PA experienced an increase in the number of friends, frequency of meetings with friends, and participation in sports and hobby clubs. Conversely, the social relationships of nonrecipients in 2013 who started to receive PA in 2016 did not significantly change.
Nishioka D, et al. ^(53)^	2022	Retrospective cohort study	Recipients in six municipalities in Japan	Municipal database	15,739	Frequent outpatient attendance (FOA)	Using CART analyses, the employed subpopulation with mental disabilities exhibited the highest risk of FOA (incidence proportion: 16.7%). Conventional regression analyses revealed that being unemployed was significantly associated with frequent outpatient attendance (IR: 1.71, 95% CI: 1.13-2.59). Living alone was also a predictor in regression analyses but not in CART analyses.
Imamatsu Y, et al. ^(54)^	2023	Cross-sectional study	Randomly sampled older recipients in Japan	Social survey data (self-questionnaire)	1,608	Health behavior scale for older adults living alone and receiving PA (HBSO)	Recipients with higher Lubben social network scale scores have more preferable HBSO scores. A health checkup was also a predictor of a higher HBSO
Nishioka D, et al. ^(55)^	2023	Retrospective cohort study	Adult recipients in two municipalities in Japan (>=20 years old)	Municipal database	2,386	Unscheduled asthma visits	Among working recipients, the IRs of unscheduled visits were higher among recipients cohabiting with adults (IR: 1.90, 95% CI: 1.00-3.59) and recipients cohabiting with children (IR: 2.35, 95% CI: 1.11-4.95) than among recipients living alone. Among the nonworking recipients, the IRs of unscheduled visits were lower among recipients living with family (IR: 0.74, 95% CI: 0.41-1.35) and those living with children (IR: 0.50, 95% CI: 0.20-1.23) than among recipients living alone
Ueno K, et al. ^(56)^	2023	Cross-sectional study	Older recipients in two municipalities in Japan (>=65 years old)	Municipal database	3,165	Clustering older recipients using the soft clustering method	Employing a soft clustering technique can help identify meaningful segments among older recipients, which is useful in considering support measures for the recipients

### Studies using the proportion of the population receiving public assistance as an environmental predictor in ecological studies

Eight studies used the proportion of the population receiving public assistance as a variable to express environmental factors across prefectures. Among these studies, only one used the proportion of the population receiving public assistance as a community-level economic factor in a cohort study ^(60)^. This study demonstrated that among men, the risk of cancer-related death was significantly higher with an increase in the proportion of households receiving public assistance. Seven were ecological studies ^(57), (58), (59), (61), (62), (63), (64)^. Considering the nature of ecological studies, it is inappropriate to apply the evidence to individual public assistance recipients, as people living in prefectures with a higher proportion of the population receiving public assistance tend to have unfavorable health conditions. Yoshikawa et al. reported that the COVID-19 incidence and mortality rates were higher in prefectures with a high proportion of the population receiving public assistance (Table 3) ^(63)^.

**Table 3.** Descriptive Information on the Studies Using the Proportion of the Population Receiving PA as an Environmental Predictor in Ecological Studies Included in the Scoping Review.

Authors	Year	Design	Settings/data	N	Outcome	Findings
Aihara H, et al. ^(57)^	2002	Ecological study	Yearly Annual Report of Hygiene in Osaka	5 areas in Osaka City	The Standardized Mortality Ratio (SMR) of suicide (standardization to the 1980 Japanese population) in each area in Osaka	Among young (<40 years old) and middle-aged (40-64 years old) men, the number of persons per household and the availability of PA were associated with a higher SMR of suicide Among middle-aged (40-64 years old) women, the suicide rate was associated with the number of PA recipients per 1,000 persons for the period between 1980 and 1999 A real difference in association between the number of persons per household, the PA, and SMR in suicide was observed in Osaka prefecture
Aihara H, et al. ^(58)^	2003	Ecological study	Yearly vital statistics for Japan	47 prefectures	The SMR of suicide (standardization to the 1980 Japanese population) in each prefecture	Multivariate regression analyses revealed that the male SMR of suicide was negatively associated with the number of persons per household and PA
Mishina H, et al. ^(59)^	2012	Ecological study	Data from home visits in the area covered by Fushimi Health Center to 2,933 mothers who had given birth between December 1, 2008 and October 31, 2010	5 localities in Fushimi-ward in Kyoto City	The detection rate of postpartum depressive symptoms in mothers	The proportion of households receiving PA in the localities had a positive correlation with the detection rate of postpartum depressive symptoms (r = 0.90, *P* = 0.04)
Honjo K, et al. ^(60)^	2014	A retrospective cohort study	Data from the Japan Collaborative Cohort Study for Evaluation of Cancer Risk (JACC Study), followed up from 1990 to 2006 in 45 municipalities	35 municipalities	All-cause death, cardiovascular death, cancer death, and death from injury, toxicosis, or external causes	Among men, the risk of death from cancer was significantly higher in the prefecture with a higher proportion of households receiving PA (coefficient 0.150 with SE 0.063 per unit increase)
Minagawa Y, et al. ^(61)^	2017	Ecological study	Prefectural data from a Comprehensive Survey of Living Conditions (Kokumin Seikatsu Kiso Chosa) in 2010	47 prefectures	Prefecture-specific disability-free life expectancy (DFLE) at 65 years of age in 2010	The percentage of older people receiving PA in prefecture was negatively associated with DFLE at 65 for women only (men: −0.01, not significant; women: −0.30, *p* < 0.001 per 1% point increase)
Okui T, et al. ^(62)^	2021	Ecological study	Prefectural data was aggregated from individual data in the National Database of Health Insurance Claims and Specific Health Checkups of Japan (NDB) data in the period of 2015-2018	47 prefectures	Standardized claim ratio (SCR) of the number of multidrug prescriptions (number of simultaneous prescriptions of seven or more internal medicines) by prefectures	The number of PA recipients per 1,000 persons was positively and significantly associated with the SCR (standardized partial regression coefficient = 0.244, *p* = 0.038)
Yoshikawa Y, et al. ^(63)^	2021	Ecological study	The data on the cumulative number of cases with positive COVID-19 test results and deaths at the prefectural and national levels was provided by the Ministry of Health, Labour and Welfare of Japan	47 prefectures	Rate ratios of COVID-19 incidence and mortality across prefectures	In prefectures with the most socioeconomic disadvantages, indicated by a greater proportion of the population receiving PA, there were higher incidence (RRs 2.45, 95% CI: 2.43-2.48) and mortality (RRs 2.02, 95% CI: 1.88-2.18)
Okui T, et al. ^(64)^	2022	Ecological study	Prefectural data aggregated from individual data in the National Database of Health Insurance Claims and Specific Health Checkups of Japan (NDB) data in the period of 2015-2018	47 prefectures	SCR of the amount of diazepam, equivalent dose prescriptions by prefectures	The number of PA recipients per capita was positively and significantly associated with the SCR (0.296, 95% CI: 0.0072-0.522) in hypnotics and (0.284, 95% CI: 0.096-0.476) in anxiolytics by a unit increase

PA, public assistance

## Discussion

To the best of our knowledge, this was the first review to describe the health status of impoverished individuals receiving public assistance in Japan. The review included 56 studies, and we elucidated that the available evidence on the health status of public assistance recipients was based on observational data, including national and governmental data, social surveys, and hospital-based registration data. We found that public assistance recipients tended to have a more disadvantageous health and well-being statuses than the general population and that the prevalence of health outcomes varied across prefectures among the recipients. Focusing on people receiving public assistance, sociodemographic inequalities in health outcomes across recipients’ characteristics, such as household composition and employment status, were found. The proportion of the population receiving public assistance may be available as a prefectural monitoring measure or as an adjustment measure of community-level economic factors in public health studies in Japan.

As mentioned, descriptions of the health statuses of public and social assistance recipients have globally accumulated in recent years. Global evidence, excluding the Japanese population, has indicated that recipients have poorer health outcomes and problematic health behaviors than the general population not receiving public or social assistance ^(12), (69), (70)^. The present review identified that the health status of public assistance recipients in Japan was consistent with previous global evidence; thus, global evidence may also be applicable to these recipients. Shahidi et al. suggested that the use of public/social assistance programs may not be sufficiently protective for the health of impoverished individuals ^(12), (69)^. From the perspective of multidimensional poverty ^(11)^, additional social support beyond financial support for public assistance recipients in Japan may also be necessary to maintain the health of recipients.

The studies included in this review have several strengths and limitations. First, studies that utilized public assistance as an exposure variable focused on marginalized populations in which ordinary social surveys could not have been sufficiently outreached. However, these studies could only capture potential associations between the receipt of public assistance and health outcomes. Second, evidence based on studies using public assistance as a study population that used national and local governmental data had the strength of analyzing complete demographic data with few missing data; however, there were limitations in the generalizability of the findings due to the limited number of municipalities included in the review. Furthermore, the evidence included in this study indicates that public assistance recipients have more undesirable health risks than the general population, and a missing link regarding the mechanism that produces health inequality across the population remains unknown. When compared between public assistance recipients and nonrecipients, several studies have shown controversial results, with inverse or no association for health outcomes ^(14), (17), (18), (28), (32), (34)^. This may be due to the effect of the public assistance as social security on access to health care and the effect of social welfare support related to the public assistance system. However, each study is specific to its target population, and the results are extremely limited, requiring further studies with diverse population to address the underlying mechanisms. In addition, as public assistance recipients differ from the general population in their characteristics and the social context leading to their receipt of public assistance, studies that remove these biases and estimate causal relationships are needed.

More detailed socioeconomic status information on the daily lifestyles of public assistance recipients and longitudinal observations are needed to elucidate the mechanisms underlying the health problems of recipients. This may enable us to make more precise causal inferences regarding the modifying effects of public assistance on the relationship between poverty and health.

This review had several strengths and limitations. The results of this review were based on currently available robust evidence from peer-reviewed papers following a comprehensive literature search. However, we restricted our search to English-language publications. English-language publications have mainly been published in the last decade, and evidence may not have been sufficiently accumulated. Furthermore, the authors acknowledge the importance of reviewing Japanese-language publications; Japanese-language publications that quantitatively described the health and well-being statuses of the recipients were sparse ^(71), (72), (73), (74), (75)^. However, evidence from Japanese publications was consistent with the findings of this review and did not alter our conclusions.

In conclusion, public assistance recipients in Japan face more health risks than the general population, and the distribution of the risks is heterogeneous among recipients despite the minimum income protection and financial benefits in health and long-term care use. Further studies are warranted to identify the effect of public assistance policies on the impoverished population and to strategize effective support for recipients’ health. Evidence-based discussions and reforms of social security policies are imperative to protect individuals’ healthy lives, even when they fall into poverty.

## Article Information

This article is based on the study, which received the Medical Research Encouragement Prize of The Japan Medical Association in 2023.

### Conflicts of Interest

None

### Sources of Funding

This work was supported by [the Japan Society for the Promotion of Science KAKENHI] grant number [22K17404] and the [Health Labor Sciences Special Research Grant] grant number [23CA2001]. This work was supported by the Medical Research Encouragement Prize of The Japan Medical Association.

### Acknowledgement

We thank Editage (www.editage.com) for the English language editing.

### Author Contributions

KH and DN conceptualized and designed the study. Both authors searched relevant literature and prepared the manuscript. All authors contributed equally to this study and approved the final version of the manuscript.

### Approval by Institutional Review Board (IRB)

Not applicable.
